# Editorial (Preface) for the Special Issue on Advances in Minimally Invasive Liver Resection for Cancer Therapies

**DOI:** 10.3390/cancers15133520

**Published:** 2023-07-06

**Authors:** Zenichi Morise

**Affiliations:** Department of Surgery, School of Medicine, Fujita Health University Okazaki Medical Center, 1 Gotanda Harisakicho, Okazaki 444-0827, Aichi, Japan; zmorise@fujita-hu.ac.jp; Tel.: +81-564-64-8800; Fax: +81-564-64-8135

After the initial reports of laparoscopic liver resection (LLR) in the early 1990s, minimally invasive liver resection has been rapidly developing based on technical and instrumental improvements [1] during its first 30 years, with two international consensus conferences [2,3] and three world congresses of the International Laparoscopic Liver Society [4]. Resections in the anterolateral segments and left lateral sectionectomy were established as common surgical procedures. Laparoscopic hemi-hepatectomies and sectionectomies (excluding left lateral sectionectomy), handling straightforward caudal–cranial transection planes suitable for the laparoscopic approach, followed them [1,3]. Partial resections and segmentectomies in posterosuperior segments (segments 1, 4a, 7, and 8), repeat LLR, and various untypical anatomical resections (such as extended anatomical resections, combinations of small anatomical resections, and hepatic-vein-guided resections, with or without preoperative simulations and intraoperative navigations) are now on their way to being established as generalized practices that many centers can adapt. Many attempts to conquer its specific disadvantages, such as the lack of a 3D view, movement restriction, little tactile sensation, and difficulty to obtain a good overview for the whole operative field, were performed. Thereafter, almost all styles of LLR without vessel reconstruction can be currently performed in many centers. However, the difficulty leading to open conversion and morbidity/mortality is different in each specific case. It not only depends on the resection style but also tumor condition (size/number/location/proximity to major vessels) as well as a patient’s general condition (performance status, comorbidities, etc.) and liver condition (such as background chronic liver diseases (CLDs) in hepatocellular carcinoma (HCC) patients and post-chemotherapy liver damage in patients with colorectal liver metastasis (CRCLM)). For these situations, several difficulty scoring systems (DSSs) have been developed for patient selection and the safe dissemination of procedures based on a learning curve.

During these developments, not only the feasibility after conquering disadvantages but also specific advantages were discussed. Less intraoperative blood loss, less morbidity, and shorter hospital stays with comparable long-term outcomes have been generally reported for HCC and CRCLM [5,6,7]. We reported the novel concept of a “caudal approach to LLR” in 2013 [8], which was defined as a main conceptual change from open liver resection to LLR in the statement of the 2nd International Consensus Conference on LLR [3]. We reported that this LLR-specific approach can cause the benefits of LLR for CLD patients who sometimes develop postoperative liver failure and often need repeated treatments for multifocal and metachronous HCC [5,9]. The basic approach of LLR, the “caudal approach”, can make minimum manipulation (damage) of the residual liver and surrounding structures (such as collateral vessels in CLD patients) possible, and leads to less liver-related morbidity/mortality plus less deterioration of liver function after liver resection. Similarly, repeat liver resection can be performed with minimum adhesiolysis in the approach, with the benefits of less blood loss, less morbidity, and shorter hospital stays with comparable operation times and long-term outcomes [10] (Figure 1).

Liver resection is a procedure in which the liver protected inside a subphrenic “rib cage” is handled and resected. The directions of view and manipulation in each approach are indicated with red arrows. (A) In open liver resection, the cage is opened with a big subcostal incision followed by lifting of the costal arch, and the mobilized liver is picked up from the retroperitoneum. (B) In the laparoscopic approach, the instruments were introduced into the cage from the caudal direction and the surgery was performed with minimal damage to the associated structures.

This field is still developing. LLR for cancers has been mainly applied for the patients with HCC and CRCLM as curative-intent resection [12]. LR for each disease has its own specificity based on disease characteristics and background liver condition. HCC patients mostly with a CLD background often develop postoperative liver failure and multifocal metachronous HCCs that need repeated treatment of liver resection in combination with (sometimes as a salvage therapy for) RFA/TACE during their long-term treatment histories. For those patients, LLR is now applied for its advantages. Anatomical resection is recommended for the disease due to its feature of spreading through the portal vein system. Precise anatomical LLR using ICG staining, etc., is developing. CRCLM patients often have postchemotherapy liver injury and multiple tumors. LLR could be used for fragile and congestive livers, with its merit of less bleeding. Multiple tumors need intraoperative precise tumor-localization as well as pre- and intraoperative precise planning for the extent of resections. The localization of tumors by using ICG fluorescence in LLR is spreading. In order to expand the indication of liver resection for multiple CRCLM, two-stage hepatectomy, future remnant liver hypertrophy with portal vein embolization, and associated liver partition with portal vein ligation for staged hepatectomy have been introduced. Multiple parenchymal-sparing resections are also performed. For liver resections with these procedures, reports of LLR application are increasing. Furthermore, the early introduction of adjuvant chemotherapy after LLR with early recovery may lead to better long-term outcomes. It is an important topic.

Biliary tract carcinoma (BTC) is also one of the candidates for LLR application [12]. However, the surgery for BTC needs lymph node dissection and bile duct resection plus reconstruction. Although there are reports of LLR for peripheral intrahepatic cholangiocarcinoma, which is often treated like HCC, and gall bladder carcinoma without the need of bile duct resection, the surgeries for the other BTCs with the needs of liver resection plus lymph node dissection/bile duct resection are currently in their developing stage. Recently emerging robot-assisted LLR could work with advantages in those cases, besides complicated resections for other tumors.

Based on the above-mentioned current status, world-famous prominent teams of researchers and surgeons wrote papers on topics in which they are interested. This Special Issue, “Advances in Minimally Invasive Liver Resection for Cancer Therapies”, is dedicated to the further steps that should be taken toward implementing minimally invasive liver resection as a standard surgical practice of cancer therapy.

## Figures and Tables

**Figure 1:**
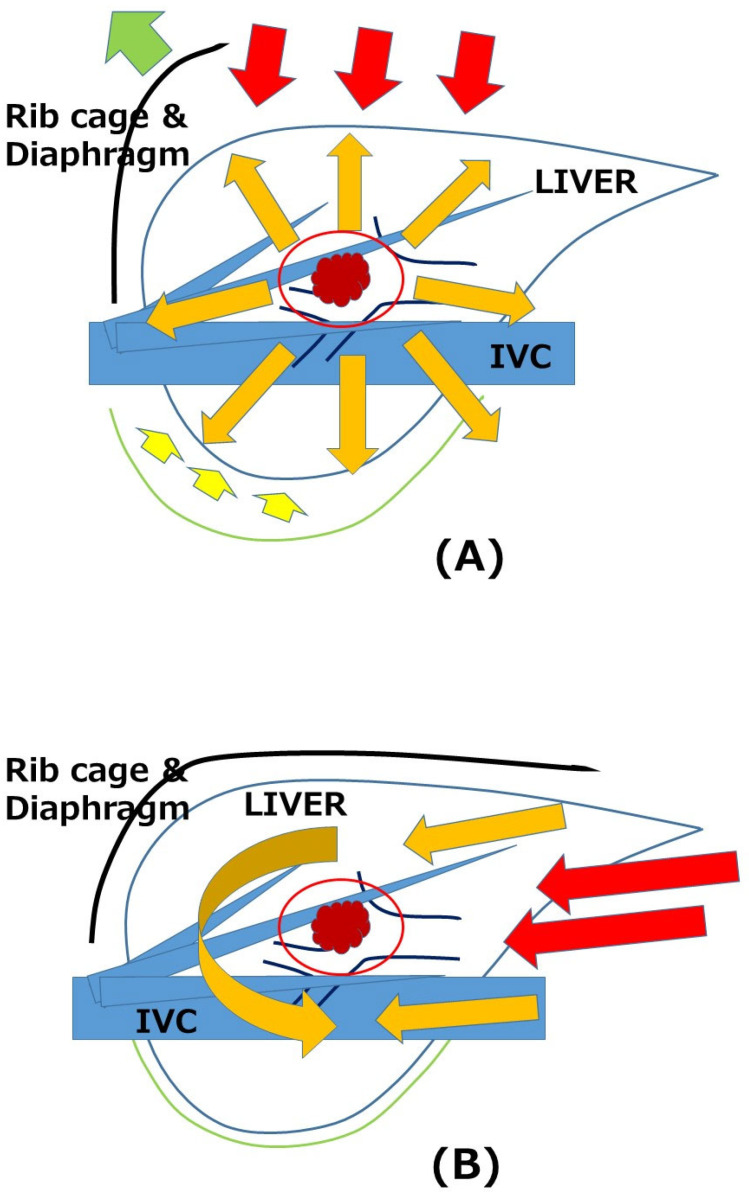
Open (**A**) and laparoscopic “caudal approach” (**B**) repeat liver resections [11].

## References

[1] Morise Z., Wakabayashi G. (2017). First quarter century of laparoscopic liver resection. World J. Gastroenterol..

[2] Buell J.F., Cherqui D., Geller D.A., O’Rourke N., Iannitti D., Dagher I., Koffron A.J., Thomas M., Gayet B., Han H.S. (2009). The international position on laparoscopic liver surgery: The Louisville Statement, 2008. Ann. Surg..

[3] Wakabayashi G., Cherqui D., Geller D.A., Buell J.F., Kaneko H., Han H.S., Asbun H., O’Rourke N., Tanabe M., Koffron A.J. (2015). Recommendations for laparoscopic liver resection: A report from the second international consensus conference held in Morioka. Ann. Surg..

[4] Cherqui D., Wakabayashi G., Geller D.A., Buell J.F., Han H.S., Soubrane O., O’Rourke N. (2016). International Laparoscopic Liver Society. The need for organization of laparoscopic liver resection. J. Hepatobiliary Pancreat. Sci..

[5] Morise Z., Ciria R., Cherqui D., Chen K.H., Belli G., Wakabayashi G. (2015). Can we expand the indications for laparoscopic liver resection? A systematic review and meta-analysis of laparoscopic liver resection for patients with hepatocellular carcinoma and chronic liver disease. J. Hepatobiliary Pancreat. Sci..

[6] Takahara T., Wakabayashi G., Beppu T., Aihara A., Hasegawa K., Gotohda N., Hatano E., Tanahashi Y., Mizuguchi T., Kamiyama T. (2015). Long-term and perioperative outcomes of laparoscopic versus open liver resection for hepatocellular carcinoma with propensity score matching: A multi-institutional Japanese study. J. Hepatobiliary Pancreat. Sci..

[7] Beppu T., Wakabayashi G., Hasegawa K., Gotohda N., Mizuguchi T., Takahashi Y., Hirokawa F., Taniai N., Watanabe M., Katou M. (2015). Long-term and perioperative outcomes of laparoscopic versus open liver resection for colorectal liver metastases with propensity score matching: A multi-institutional Japanese study. J. Hepatobiliary Pancreat. Sci..

[8] Tomishige H., Morise Z., Kawabe N., Nagata H., Ohshima H., Kawase J., Arakawa S., Yoshida R., Isetani M. (2013). Caudal approach to pure laparoscopic posterior sectionectomy under the laparoscopy-specific view. World J. Gastrointest. Surg..

[9] Berardi G., Morise Z., Sposito C., Igarashi K., Panetta V., Simonelli I., Kim S., Goh B.K.P., Kubo S., Tanaka S. (2020). Development of a nomogram to predict outcome after liver resection for hepatocellular carcinoma in Child-Pugh B cirrhosis. J. Hepatol..

[10] Morise Z., Aldrighetti L., Belli G., Ratti F., Belli A., Cherqui D., Tanabe M., Wakabayashi G., ILLS-Tokyo Collaborator group (2020). Laparoscopic repeat liver resection for hepatocellular carcinoma: A multicentre propensity score-based study. Br. J. Surg..

[11] Morise Z., Katsuno H., Kikuchi K., Endo T., Matsuo K., Asano Y., Horiguchi A. (2023). Laparoscopic Repeat Liver Resection—Selecting the Best Approach for Repeat Liver Resection. Cancers.

[12] Morise Z. (2022). Current status of minimally invasive liver surgery for cancers. World J. Gastroenterol..

